# New Coleoptera records from New Brunswick, Canada: Megalopodidae and Chrysomelidae

**DOI:** 10.3897/zookeys.179.2625

**Published:** 2012-04-04

**Authors:** Reginald P. Webster, Laurent LeSage, Ian DeMerchant

**Affiliations:** 1 Natural Resources Canada, Canadian Forest Service - Atlantic Forestry Centre, 1350 Regent St., P.O. Box 4000, Fredericton, NB, Canada E3B 5P7; 2 Canadian National Collection of Insects, Arachnids, and Nematodes, Agriculture and Agri-Food Canada, 960 Carling Avenue, Ottawa, Ontario, K1A 0C6, Canada

**Keywords:** Chrysomelidae, Megalopodidae, new records, Canada, New Brunswick

## Abstract

*Zeugophora varians* Crotch and the family Megalopodidae are newly recorded for New Brunswick, Canada. Twenty-eight species of Chrysomelidae are newly recorded for New Brunswick, including *Acalymma gouldi* Barber, *Altica knabii* Blatchley, *Altica rosae* Woods, *Altica woodsi* Isely, *Bassareus mammifer* (Newman), *Chrysolina marginata* (Linnaeus), *Chrysomela laurentia* Brown, *Crepidodera violacea* Melsheimer, *Cryptocephalus venustus* Fabricius, *Neohaemonia melsheimeri* (Lacordaire), *Neohaemonia nigricornis* (Kirby), *Pachybrachis bivittatus* (Say), *Pachybrachis m-nigrum* (Melsheimer), *Phyllobrotica limbata* (Fabricius), *Psylliodes affinis* (Paykull), *Odontota dorsalis* (Thunberg), *Ophraella communa* (LeSage), *Ophraella cribrata* (LeConte), *Ophraella notata* (Fabricius), *Systena hudsonias* (Forster), *Tricholochmaea ribicola* (Brown), and *Tricholochmaea rufosanguinea* (Say), which are also newly recorded for the Maritime provinces. Collection data, habitat data, and distribution maps are presented for all these species.

## Introduction

This paper treats the families Chrysomelidae and Megalopodidae. The Megalopodidae (megalopodid leaf beetles), historically considered a subfamily of Chrysomelidae (Seeno and Wilcox 1982), is a small family of leaf-feeding beetles related to the Chrysomelidae. Only the genus *Zeugophora* occurs in North America. Known hosts of North American species include *Populus* and *Salix* spp. Larvae are leaf miners and adults feed externally on leaves (Clark and Riley 2002). Seven species (as subfamily Zeugophorinae in the Chrysomelidae) were reported from Canada by LeSage (1991). No species were reported from this family from New Brunswick or the Maritime provinces (New Brunswick, Nova Scotia, Prince Edward Island).

The Chrysomelidae (the leaf beetles) is one of the largest families of beetles. The Chrysomelidae, as the common name implies, are phytophagous and feed on leaves of plants, usually Angiospermae. Adults of most species are either monophagous or oligophagous and usually use terrestrial species, whereas the larvae have more diverse feeding habits. Donaciinae larvae are aquatic and live on submerged stems and roots of their host (Hoffman 1940). Case-bearing larvae are found in three subfamilies in Canada: larvae of the Clytrinae feed on debris in ant nests (LeSage and Stiefel 1996), larvae of the Cryptocephalinae feed on decaying leaves in litter (LeSage 1985, 1986a), and larvae of the Chlamisiinae eat fresh leaves in the open like the adults (LeSage 1982). Root miners are mainly found in Alticini, Eumolpinae, and Galerucini, whereas leaf miners are numerous in Alticini and in all Hispini (Lawson 1991). Riley et al. (2002) presented a general review of the Chrysomelidae of North America, and that publication should be consulted for details on the classification and a general overview of the biology of members of this family.

Riley et al. (2003) reported 139 species of Chrysomelidae from New Brunswick, Canada in their catalog of the leaf beetles of North America. Since that publication, the adventive *Oulema melanopus* (Linnaeus) and *Pyrrhalta viburni* (Paykull) have been newly reported from the province by Finnamore (1988) and Weston and Hoebeke (2003), respectively. Majka and LeSage (2007) reported on the overall distribution of *Pyrrhalta viburni* in Maritime provinces, and LeSage et al. (2007) on that of *Oulema melanopus*. The following year, Majka and LeSage (2008a) reported the presence of *Chrysolina staphylaea* (Linnaeus) in Nova Scotia and Quebec, but did not report it from New Brunswick, although it will likely be found in the province with additional sampling. Majka and LeSage (2008b) and Majka and Kirby (2011) reported on the distribution and range expansion of the adventive *Lilioceris lilii* (Scopoli) in the Maritime provinces, including New Brunswick. LeSage et al. (2008) confirmed the presence of both introduced asparagus leaf beetles (*Crioceris asparagi* (Linnaeus), *Crioceris duodecimpunctata* (Linnaeus)) in the Maritimes, including New Brunswick. Majka and LeSage (2008c) confirmed the presence of the introduced *Cassida rubiginosa* Müller in New Brunswick, and the following year LeSage and Majka (2009) confirmed the presence of the introduced *Gastrophysa polygoni* Linnaeus. Most recently, Majka and LeSage (2010) reported *Chaetocnema borealis* White and *Chaetocnema protensa* LeConte from New Brunswick in their review of the *Chaetocnema* of the Maritime provinces, increasing the number of species of Chrysomelidae known from New Brunswick to 143.

A few comments are required regarding *Crepidodera digna* Parry, *Dibolia penstemonis* Parry, and *Diachus catarius* (Suffrian) which were recorded from New Brunswick by LeSage (1991) but not listed by Riley et al. (2003) in their catalog. There are no specimens of *Crepidodera digna* and *Dibolia penstemonis* in the CNC (Canadian National Collection of Insects, Arachnids, and Nematodes) and these two species were not reported from New Brunswick by Riley et al. (2003), and thus these species are excluded from the provincial list, although it is probable that both species occur in the province. There are many specimens in the CNC under the name *Dibolia catarius* but their determinations have not been verified and *Crepidodera catarius* may be a synonym of *Diachus auratus* (Fabricius). The genus *Diachus* is in need of revision. This species is therefore excluded from the provincial list until this genus is revised and the species name of the specimens in the CNC can be verified.

Intensive collecting by the first author and others since 2003 has resulted in the discovery of additional species of Chrysomelidae from New Brunswick (Table 1). Additional records were discovered in the older material preserved in the Canadian National Collection in Ottawa, including the first record of the family Megalopodidae. The purpose of this paper is to report on these new discoveries.

## Methods and conventions

The following records are based in part on specimens collected as part of a general survey by the first author to document the Coleoptera fauna of New Brunswick. A description of the habitat was recorded for all specimens collected during this survey. Locality and habitat data are presented exactly as on labels for each record. This information, as well as additional collecting notes, is summarized and discussed in the collection and habitat data section for each species.

### Collection methods

Most specimens were collected by sweeping vegetation in various habitats, and beating, sweeping, or hand picking beetles from host plants. Additional records were obtained from specimens contained in the collection belonging to Natural Resources Canada, Canadian Forest Service - Atlantic Forestry Centre, Fredericton, New Brunswick and the Canadian National collection, Ottawa, Ontario.

### Specimen preparation

Males and females of some species were dissected to confirm their identity. Male aedeagi were dissected in 70% ethanol and glued on tip of small points under the specimens from which they originated. The female genital structures were dissected in 70% ethanol, dehydrated in absolute alcohol, transferred into cedar oil, and mounted in Canada balsam on small transparent acetate cards pinned with the specimens from which they originated.

### Distribution

Distribution maps, created using ArcMap and ArcGIS, are presented for each species in New Brunswick. Every species is cited with current distribution in Canada and Alaska, using abbreviations for the state, provinces, and territories. New records for New Brunswick are indicated in bold under Distribution in Canada and Alaska. The following abbreviations are used in the text:

**Table:** 

**AK**	Alaska	**MB**	Manitoba
**YT**	Yukon Territory	**ON**	Ontario
**NT**	Northwest Territories	**QC**	Quebec
**NU**	Nunavut	**NB**	New Brunswick
**BC**	British Columbia	**PE**	Prince Edward Island
**AB**	Alberta	**NS**	Nova Scotia
**SK**	Saskatchewan	**NF & LB**	Newfoundland and Labrador

Acronyms of collections examined or where specimens reside referred to in this study are as follows:

**AFC** Atlantic Forestry Centre, Natural Resources Canada, Canadian Forest Service, Fredericton, New Brunswick, Canada

**CGMC** Christopher G. Majka Collection, Halifax, Nova Scotia, Canada

**CNC** Canadian National Collection of Insects, Arachnids, and Nematodes, Agriculture and Agri-Food Canada, Ottawa, Ontario, Canada

**NBM** New Brunswick Museum, Saint John, New Brunswick, Canada

**RWC** Reginald P. Webster Collection, Charters Settlement, New Brunswick, Canada

**UMNB **Université de Moncton Collection, Moncton, New Brunswick, Canada

## Results

### Species accounts

All records below are species newly recorded for New Brunswick, Canada. Species followed by ** are newly recorded from the Maritime provinces of Canada.

The classification of the Chrysomelidae and Megalopodidae follows Riley et al. (2003).

**Table 1.** Species of Megalopodidae and Chrysomelidae recorded from New Brunswick, Canada.

**Family Megalopodidae Latreille**
**Subfamily Zeugophorinae Böving & Craighead**
*Zeugophora varians* Crotch**
**Family Chrysomelidae Latreille**
**Subfamily Donaciinae Kirby**
**Tribe Plateumarini Askevold**
*Plateumaris balli* Askevold
*Plateumaris flavipes* (Kirby)
*Plateumaris frosti* (Schaeffer)
*Plateumaris fulvipes* (Lacordaire)
*Plateumaris germari* (Mannerheim)
*Plateumaris metallica* (Ahrens)
*Plateumaris nitida* (Germar)
*Plateumaris pusilla* (Say)
*Plateumaris rufa* (Say)
*Plateumaris shoemakeri* (Schaeffer)
**Tribe Donaciini Kirby**
*Donacia palmata* (Olivier)
*Donacia piscatrix* Lacordaire
*Donacia proxima* Kirby
*Donacia caerulea* Olivier
*Donacia confluenta* Say
*Donacia fulgens* LeConte
*Donacia hirticollis* Kirby
*Donacia magnifica* J. L. LeConte
*Donacia subtilis* Kunze
*Donacia tuberculifrons* Schaeffer
**Tribe Haemoniini Chen**
*Neohaemonia melsheimeri* (Lacordaire)**
*Neohaemonia nigricornis* (Kirby)**
**Subfamily Criocerinae Latreille**
**Tribe Criocerini Latreille**
*Crioceris asparagi* (Linnaeus)
*Crioceris duodecimpunctata* (Linnaeus)
*Lilioceris lilii* (Scopoli)
**Tribe Lemini Heinzen**
*Lema puncticollis* Curtis
*Oulema melanopus* (Linnaeus)
**Subfamily Cassidinae Gyllenhal**
**Tribe Chalepini Weise**
*Anisostena nigrita* (Olivier)
*Baliosus nervosus* (Panzer)
*Glyphuroplata pluto* (Newman)
*Microrhopala excavata excavata* (Olivier)
*Microrhopala vittata* (Fabricius)
*Microrhopala xerene* (Newman)
*Odontota dorsalis* (Thunberg)**
*Sumitrosis inaequalis* (Weber)
*Sumitrosis rosea* (Weber)
**Tribe Cassidiini Gyllenhal**
*Cassida rubiginosa* Müller
*Charidotella purpurata* (Boheman)
*Charidotella sexpunctata bicolor* (Fabricius)
*Deloyala guttata* (Olivier)
*Plagiometriona clavata clavata* (Fabricius)
**Subfamily Chrysomelinae Latreille**
**Tribe Chrysomelini Latreille**
**Subtribe Gonioctenina Motschulski**
*Gonioctena americana* (Schaeffer)
**Subtribe Doryphorina Motschulski**
*Chrysolina hyperici hyperici* (Forster)
*Chrysolina marginata* (Linnaeus)**
*Chrysolina quadrigemina* (Suffrian)
*Calligrapha bidenticola* Brown
*Calligrapha californica coreopsivora* Brown
*Calligrapha alni* Schaeffer
*Calligrapha alnicola* Brown
*Calligrapha confluens* Schaeffer
*Calligrapha ignota* Brown
*Calligrapha multipunctata* (Say)
*Calligrapha philadelphica* (Linnaeus)
*Calligrapha rowena* Knab
*Calligrapha tiliae* Brown
*Calligrapha vicina* Schaeffer
*Calligrapha virginea* Brown
*Calligrapha lunata* (Fabricius)
*Labidomera clivicollis* (Kirby)
*Leptinotarsa decemlineata* (Say)
**Subtribe Chrysomelina Latreille**
*Chrysomela crotchi* Brown
*Chrysomela laurentia* Brown**
*Chrysomela lineatopunctata* Forster
*Chrysomela mainensis mainensis* J. Bechyné
*Gastrophysa polygoni* (Linnaeus)
*Phaedon armoraciae armoraciae* (Linnaeus)
*Phaedon laevigatus* (Duftschmid)
*Phaedon oviformis* (LeConte)
*Phaedon viridis* Melsheimer
*Phratora americana canadensis* Brown
*Phratora purpurea purpurea* Brown
*Plagiodera versicolora* (Laicharting)
*Prasocuris vittata* (Olivier)*
**Subfamily Galerucinae Latreille**
**Tribe Galerucini Latreille**
*Erynephala maritima* (LeConte)*
*Galerucella nymphaeae* (Linnaeus)
*Neogalerucella calmariensis* (Linnaeus)*
*Neogalerucella pusilla* (Duftschmid)
*Ophraella conferta* (LeConte)
*Ophraella communa* (LeSage)**
*Ophraella cribrata* (LeConte)**
*Ophraella notata* (Fabricius)**
*Pyrrhalta viburni* (Paykull)
*Tricholochmaea alni* (Fall)
*Tricholochmaea cavicollis* ( LeConte)
*Tricholochmaea decora decora* (Say)
*Tricholochmaea kalmiae* (Fall)
*Tricholochmaea perplexa* (Fall)
*Tricholochmaea ribicola* (Brown)**
*Tricholochmaea rufosanguinea* (Say)**
*Tricholochmaea tuberculata* (Say)
*Tricholochmaea vaccinii* (Fall)
*Trirhabda borealis* Blake
*Trirhabda canadensis* (Kirby)
*Trirhabda virgata* LeConte
*Xanthogaleruca luteola* (Müller)
**Tribe Luperini Chapuis**
**Subtribe Diabroticina Chapuis**
*Acalymma vittatum* (Fabricius)
*Acalymma gouldi* Barber**
*Diabrotica barberi* R. Smith & Lawrence
**Subtribe Luperina Chapuis**
*Phyllobrotica decorata* (Say)
*Phyllobrotica limbata* (Fabricius)**
*Scelolyperus cyanellus* (LeConte)
*Scelolyperus meracus* (Say)
**Tribe Alticini Newman**
*Altica ambiens alni* Harris
*Altica browni* Mohamedsaid
*Altica carinata* Germar
*Altica corni* Woods
*Altica kalmiae* (Melsheimer)
*Altica knabii* Blatchley**
*Altica prasina populi* Brown
*Altica rosae* Woods**
*Altica sylvia* Malloch
*Altica tombacina* Mannerheim
*Altica ulmi* Woods
*Altica woodsi* Isely**
*Capraita subvittata* (Horn)
*Chaetocnema borealis* White
*Chaetocnema concinna* (Marsham)
*Chaetocnema confinis* Crotch
*Chaetocnema minuta* Melsheimer
*Chaetocnema protensa* LeConte
*Crepidodera heikertingeri* (Lazorko)
*Crepidodera luminosa* Parry
*Crepidodera nana* (Say)
*Crepidodera populivora* Parry
*Crepidodera violacea* Melsheimer**
*Dibolia borealis* Chevrolat
*Dibolia melampyri* Parry
*Disonycha alternata* (Illiger)
*Disonycha latifrons* Schaeffer
*Disonycha xanthomelas* (Dalman)
*Distigmoptera borealis* Blake
*Distigmoptera impennata* Blake
*Epitrix cucumeris* (Harris)
*Kuschelina vians* (Illiger)
*Longitarsus erro* Horn*
*Longitarsus jacobaeae* (Waterhouse)
*Longitarsus luridus* (Scopoli)
*Longitarsus testaceus* (Melsheimer)
*Mantura chrysanthami* (Koch)*
*Phyllotreta armoraciae* (Koch)
*Phyllotreta cruciferae* (Goeze)
*Phyllotreta robusta* LeConte
*Phyllotreta striolata* (Fabricius)
*Phyllotreta zimmermanni* (Crotch)
*Psyliodes affinis* (Paykull)**
*Psyliodes cucullatus* (Illiger)
*Psyliodes napi* (Fabricius)
*Psyliodes punctulatus* Melsheimer
*Systena frontalis* (Fabricius)
*Systena hudsonias* (Forster)**
**Subfamily Eumolpinae Hope**
**Tribe Synetini**
*Syneta extorris borealis* Brown
*Syneta ferruginea* (Germar)
*Syneta pilosa* Brown
**Tribe Adoxini Baly**
*Bromius obscurus* (Linnaeus)
*Xanthonia decemnotata* (Say)
**Subfamily Cryptocephalinae Gyllenhal**
**Tribe Cryptocephalini Gyllenhal**
**Subtribe Pachybrachina Chapuis**
*Pachybrachis bivittatus* (Say)**
*Pachybrachis m-nigrum* (Melsheimer)**
*Pachybrachis peccans* Suffrian
*Pachybrachis pectoralis* (Melsheimer)
**Subtribe Monachulina Leng**
*Lexiphanes saponatus* (Fabricius)
**Subtribe Cryptocephalina Gyllenhal**
*Bassareus formosus* (Melsheimer)*
*Bassareus mammifer* (Newman)**
*Cryptocephalus gibbicollis gibbiciollis* Haldeman
*Cryptocephalus notatus* Fabricius
*Cryptocephalus venustus* Fabricius**
*Diachus auratus* (Fabricius)
*Triachus vacuus* LeConte
**Tribe Chlamisini Gressitt**
*Exema canadensis* Pierce
*Neochlamisus comptoniae* (Brown)
*Neochlamisus cribripennis* (J. L. LeConte)
*Neochlamisus eubati* (Brown)
*Neochlamisus fragariae* (Brown)

**Notes:** *New to province, **New to Maritime provinces.

### Family Megalopodidae Latrielle, 1802. Zeugophorinae Böving and Craighead, 1931

#### Zeugophora varians

Crotch, 1873**

http://species-id.net/wiki/Zeugophora_varians

Map 1

##### Material examined.

**New Brunswick, Gloucester Co.**, Tracadie, 30.VII.1939, W. J. Brown (1, CNC). **Kent Co.**, Kouchibouguac National Park, 5.VII.1977, S. J. Miller, 5786N (1, CNC); same locality, collector, and date, 5487A (1, CNC); same locality and collector, 9.VIII.1977, 5805B (1, CNC); same locality and collector, 16.VIII.1977, 6054V (2, CNC). **Saint John Co.,** Saint John, Rockwood Park, 7.VIII.1953, J. F. Brimley (1, CNC). **York Co.** Fredericton, 16.VII.1928, W. J. Brown (4, CNC).

##### Collection and habitat data.

No bionomic data were associated with the specimens. This species has been recorded from *Populus balsmifera* L., *Populus tremuloides* Michx. and *Salix* (Clark et al. 2004).

##### Distribution in Canada and Alaska.

BC, AB, SK, MB, QC, **NB** (Riley et al. 2003). These are the first records of this family for New Brunswick.

**Map 1.**
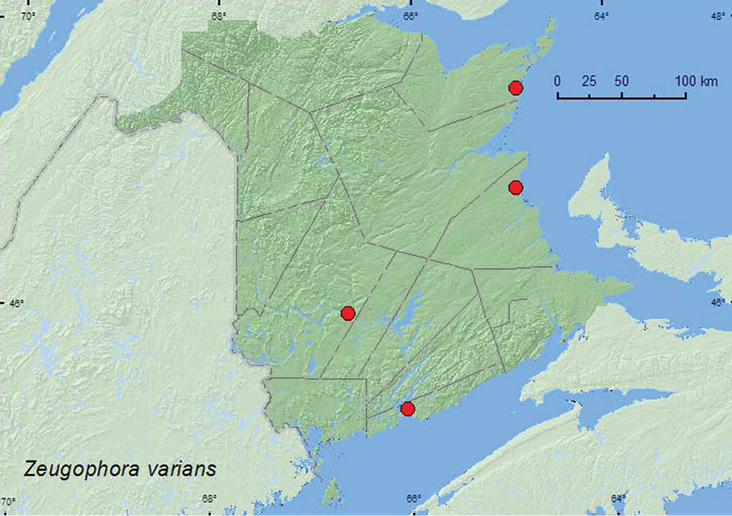
Collection localities in New Brunswick, Canada of *Zeugophora varians*.

### Family Chrysomelidae Latreille, 1802. Subfamily Donaciinae Kirby, 1837. Tribe Haemoniini Chen, 1941

#### Neohaemonia melsheimeri

(Lacordaire)**

http://species-id.net/wiki/Neohaemonia_melsheimeri

Map 2

##### Material examined.

**New Brunswick, York Co.**, Mazerolle Settlement, 45.8765°N, 66.8260°W, 8.VI.2008, R. P. Webster, beaver meadow, sweeping vegetation along brook margin (1, RWC).

##### Collection and habitat data.

This species has been collected from leaves and stems of pondweeds (*Potamogeton* sp.) (Potamogetonaceea) (Askevold 1987) and from leaf litter beside small lakes from October to the first snow (L. LeSage, personal observation). *Neohaemonia* species occur mostly in lotic sites near streams and are often submerged, and thus, are rarely collected (Askevold 1987). Larvae are submerged and feed on the stems and roots of *Potamogeton* (Hoffman 1940). The single adult from New Brunswick was collected by sweeping vegetation along a stream margin in early June.

##### Distribution in Canada and Alaska.

MB, ON, QC, **NB** (LeSage 1991).

**Map 2.**
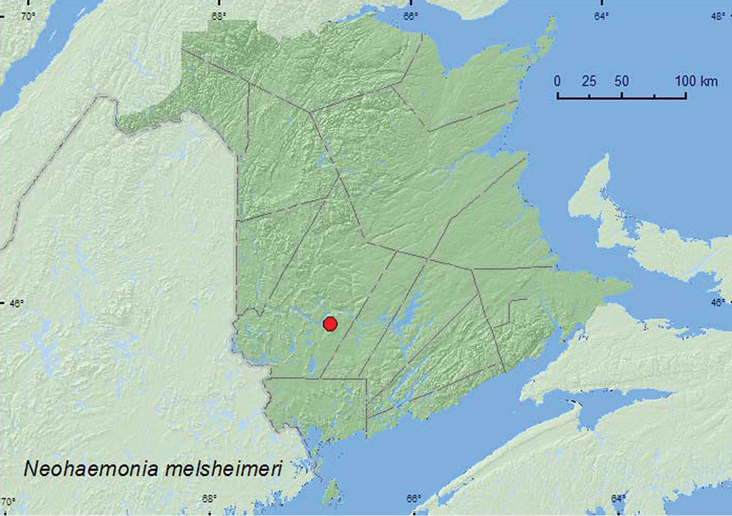
Collection localities in New Brunswick, Canada of *Neohaemonia melsheimeri*.

#### Neohaemonia nigricornis

(Kirby, 1837)**

http://species-id.net/wiki/Neohaemonia_nigricornis

Map 3

##### Material examined.

**New Brunswick, Queens Co.**, Scotchtown at Grand Lake, 45.8760°N, 66.1816°W, 25.VI.2003, R. P. Webster, lake margin, on foliage.

##### Collection and habitat data.

This species has been collected from leaves and stems of pondweeds (*Potamogeton* sp.) (Askevold 1987) and probably has a similar biology as *Neohaemonia melsheimeri*. One adult from New Brunswick was swept from foliage along a lake margin during June.

##### Distribution in Canada and Alaska

**.** BC, MB, ON, QC, **NB** (Askevold 1987).

**Map 3.**
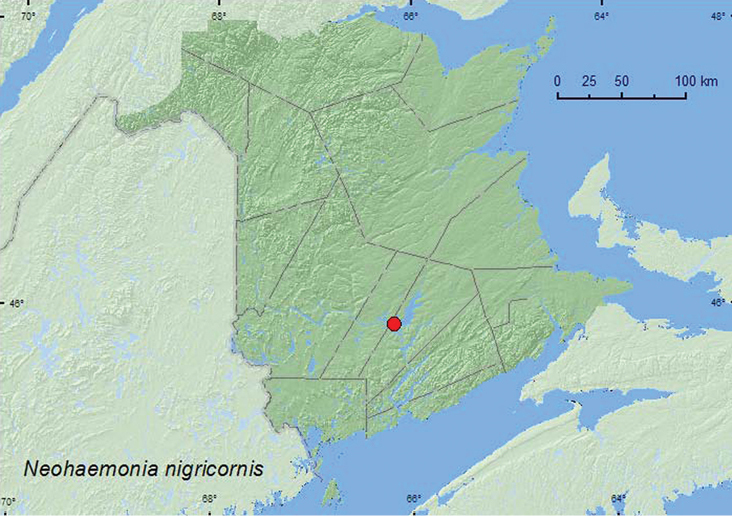
Collection localities in New Brunswick, Canada of *Neohaemonia nigricornis*.

### Subfamily Cassidinae Gyllenhal, 1813. Tribe Chalepini Weise, 1910

#### Odontota dorsalis

(Thunberg, 1805)**

http://species-id.net/wiki/Odontota_dorsalis

Map 4

##### Material examined.

**New Brunswick, Queens Co.**, Canning, near Flowers Cove off Rt. 960, 46.0363°N, 66.0387°W, 1.VII.2004, D. Sabine & R. Webster, on foliage of *Robinia pseudoacacia* L. (14, CNC, NBM, RWC). **York Co.**, Fredericton, 23.IX.2009, C. Maund, on apple trees (1, CNC).

##### Collection and habitat data.

In New Brunswick, adults were collected from foliage of black locust(*Robinia pseudoacacia* L.) in early July. One individual was collected from an apple (*Malus pumilla* P. Mill.) tree. Larvae mine the leaves of black locust and other woody species of Fabaceae. Adults also feed on black locust and other Fabaceae but have been collected from many other tree species (Clark et al. 2004; Staines 2006).

##### Distribution in Canada and Alaska.

MB,ON, QC, **NB** (LeSage 1991; Riley et al. 2003).

**Map 4.**
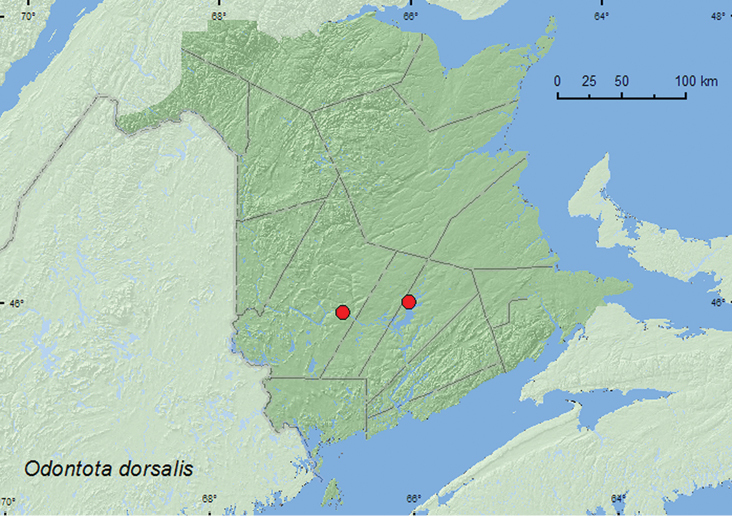
Collection localities in New Brunswick, Canada of *Odontota dorsalis*.

### Subfamily Chrysomelinae Latreille, 1802. Tribe Chrysomelini Latreille, 1802. Subtribe Doryphorina Motschulski, 1860

#### Chrysolina marginata

(Linnaeus, 1758)**

http://species-id.net/wiki/Chrysolina_marginata

Map 5

##### Material examined.

**New Brunswick, Queens Co.**, Cranberry Lake P.N.A. (Protected Natural Area), 46.1125°N, 65.6075°W, 18.VI.2009, R. Webster & M.-A. Giguère, red oak forest, sweeping foliage (in area with *Leucanthemum vulgare* Lam.) (1, AFC). **Northumberland Co.**, Blueberry Rd. off Hwy 8, 47.3211°N, 65.4223°W, 29.VI.2007, R. P. Webster, jack pine forest with black spruce, on *Leucanthemum vulgare* Lam. (1, CNC, RWC). **York Co.**, New Maryland, 45–50.50°N, 66–43.93°W, 5.IX.2002, R. P. Webster (1, CNC). Charters Settlement, 45.8395°N, 66.7391°W, 20.X.2004, 20.X.2004, 26.IX.2005, 21.X.2005, 28.IX.2006, R. P. Webster, (on pavement of street) (1, CNC, 2, RWC); 15.0 km W of Tracy off Rt. 645, 45.6837°N, 66.8809°W, 16.VI.2007, R. P. Webster, red pine forest, on *Leucanthemum vulgare* Lam. (1, CNC, 1, RWC).

##### Collection and habitat data.

 Adults from New Brunswick were collected from the foliage of *Leucanthemum vulgare* Lam. (ox-eye-daisy) in open disturbed roadside sites near a red pine (*Pinus resinosa* Ait.) and a jack pine (*Pinus banksiana* Lamb.) forest. Specimens were also collected in the late fall on a paved road during warm afternoons. Adults were collected during June, September, and October.

##### Distribution in Canada and Alaska.

 AK, YT, **NB** (Riley et al. 2003). The population in New Brunswick is likely an adventive Palaearctic species known from Europe, Siberia, the Far East, and Alaska (Bieńkowski 2001).

##### Comment.

*Chrysolina finitima* Brown, 1962 was placed in synonymy with *Chrysolina marginata marginata* (Linnaeus) by Bieńkowski (2001: 152), a synonymy accepted by Riley et al. (2003) in their catalog. It makes sense for specimens from Alaska or Yukon to belong to the nominal Palaearctic subspecies since this state and province can be considered as the easternmost part of the natural distribution of *Chrysolina marginata* that extends over the Bering Detroit into the New World. On the other hand, the presence of *Chrysolina marginata* in New Brunswick is not natural and is undoubtedly the result of a recent introduction into eastern Canada, which is not yet fully documented (LeSage, personal observations). Considering that there are nine Palaearctic subspecies (Bieńkowski 2011), it might be advisable not to use a subspecies name until our eastern population can be properly assigned to a subspecies.

**Map 5.**
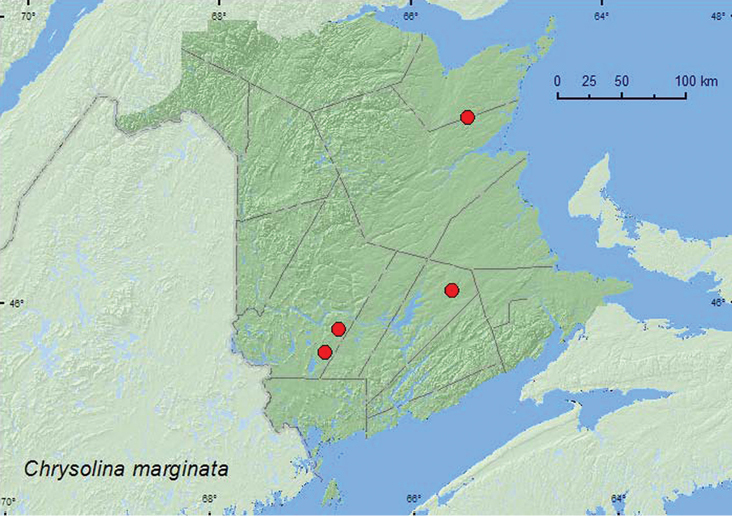
Collection localities in New Brunswick, Canada of *Chrysolina marginata*.

### Subtribe Chrysomelina Latrielle, 1802

#### Chrysomela laurentia

Brown, 1956**

http://species-id.net/wiki/Chrysomela_laurentia

Map 6

##### Material examined.

**New Brunswick, Carleton Co.**, Meduxnekeag Valley Nature Preserve, 46.1890°N, 67.6766°W, 1.VIII.2004, V. Webster & R. Webster, river margin, sweeping foliage (1, RWC); same locality but 46.1931°N, 67.6825°W, 8.VI.2005, M.-A. Giguère & R. Webster, floodplain forest, sweeping (1, RWC); same locality data, 25.VI.2007, R. P. Webster, forest near river margin, beating foliage of *Salix* sp. (1, RWC). **York Co.**, 1.5 km S of Taymouth, 46.1582°N, 66.6134°W, 15.VI.2006, R. P. Webster, Nashwaak River, on sand bar, on *Salix* sp. foliage (2, RWC). **Saint John Co.**, Saint John, 9.VI.1901, W. McIntosh (1, NBM); Saint John, VII.1901, W. McIntosh (1, NBM).

##### Collection and habitat data.

 The main host plants of *Chrysolina laurentia* are *Salix* sp., with known preferences for *Salix discolor* Mühl., *Salix interior* Mühl, *Salix lucida* Mühl., and *Salix petiolaris* J.E. Smith (LeSage 1996), but poplars (*Populus* sp.) are also accepted (Brown 1956). In New Brunswick, this species was collected by beating foliage of *Salix* or sweeping foliage along river margins. Adults were collected during June and August.

##### Distribution in Canada and Alaska.

NT, AB, ON, QC, **NB** (LeSage 1991).

**Map 6.**
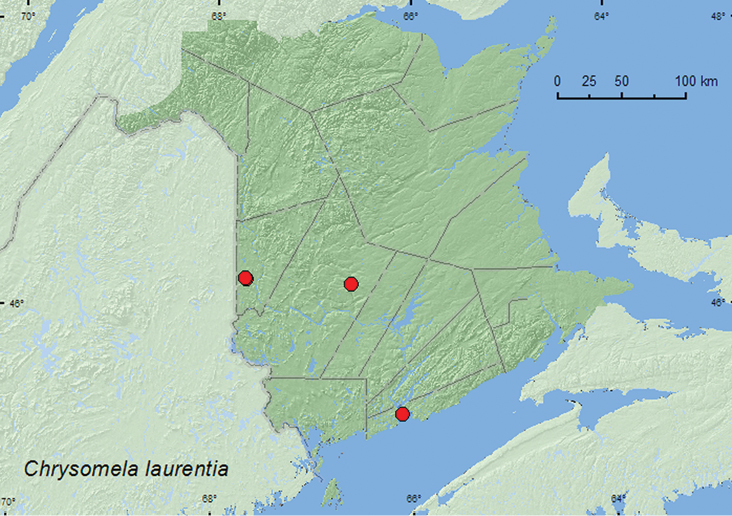
Collection localities in New Brunswick, Canada of *Chrysomela laurentia*.

#### Prasocuris vittatus

(Olivier, 1807)

http://species-id.net/wiki/Prasocuris_vittatus

Map 7

##### Material examined.

**New Brunswick, Restigouche Co.**, Jacquet River Gorge P.N.A., 47.8160°N, 65.9928°W, 25.VI.2008, R. P. Webster, mixed forest, sweeping roadside foliage (2, RWC). **Saint John Co.**, Saint John, VI.190?, W. McIntosh (1, NBM). **York Co.**, Canterbury, 45.8841°N, 67.6428°W, 8.VI.2004, D. Sabine & R. Webster, hardwood forest, sweeping foliage of small marsh (sedges) (1, RWC); same locality but 45.8972°N, 67.6272°W, 21.VII.2004, D. Sabine, J. Edsall, K. Bredin, & R. Webster, mixed forest with cedar, sweeping foliage near small stream (2, RWC); Canterbury, Browns Mtn. Fen, 45.8977°N, 67.6335°W, 1.VI.2005, M.-A. Giguère & R. Webster, mixed forest, sweeping foliage along forest trail (5, RWC).

##### Collection and habitat data.

*Prasocuris vittatus* was collected by sweeping foliage along a roadside and forest trail, in a small marsh with *Carex*, and near a small stream. However, the true host is probably buttercup (*Ranunculus acris* L.) on which both larvae and adults were found and reared by the second author. *Ranunculus acris* and *Ranunculus repens* L. (Creeping buttercup) were reported as hosts for this species by Clark et al. (2004). Adults were collected during June and July.

##### Distribution in Canada and Alaska.

 NT, AB, SK, MB, ON, QC, **NB**, NS (LeSage 1991).

**Map 8.**
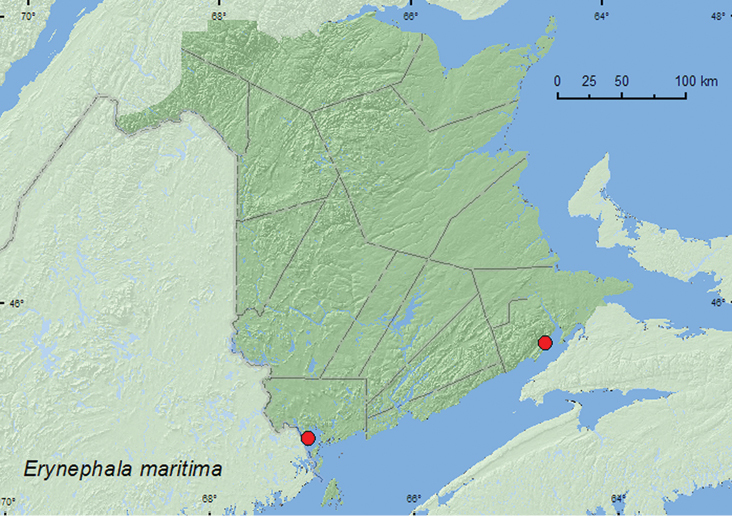
Collection localities in New Brunswick, Canada of *Erynephala maritima*.

### Subfamily Galerucinae Latreille, 1802. Tribe Galerucini Latreille, 1802

#### Erynephala maritima

(LeConte, 1865)

http://species-id.net/wiki/Erynephala_maritima

Map 8

##### Material examined.

**New Brunswick, Albert Co.**, Mary’s Point, 20.VIII.2005, C. G. Majka, salt marsh (5, CGMC). **Charlotte Co.**, St. Andrews, 45.0751°N, 67.0374°W, 25.VIII.2006, R. P. Webster, sea beach, sweeping foliage (7, RWC).

##### Collection and habitat data.

*Erynephala maritima* was swept from foliage along a sea beach in August. According to Clark et al. (2004), this species is primarily associated with various species of Chenopodiaceae (*Beta*, *Chenopodium*, *Salicornia*, *Salsola*, *Suaeda*).

##### Distribution in Canada and Alaska.

**NB**, NS (LeSage 1991).

**Map 7.**
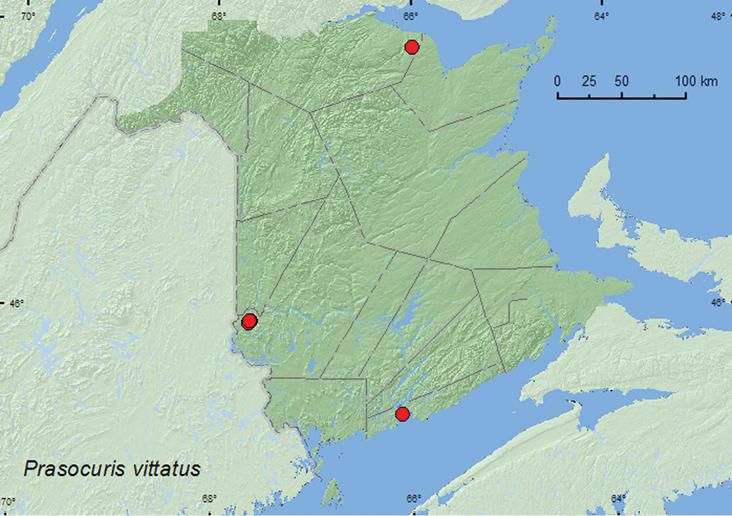
Collection localities in New Brunswick, Canada of *Prasocuris vittatus*.

#### Neogalerucella calmariensis

(Linnaeus, 1767)

http://species-id.net/wiki/Neogalerucella_calmariensis

Map 9

##### Material examined. 

**New Brunswick, Queens Co.**, Scotchtown near Indian Point, 45.8762°N, 66.1816°W, 5. VI.2004, 9.VII.2006, R. P. Webster, margin of lake, oak maple forest on sandy soil, sweeping foliage (6, NBM, RWC). **Sunbury Co.**, about 2.0 km ESE of Gilbert Island at St. John River, 45.8712°N, 66.2705°W, 26.VI.2003, R. P. Webster, silver maple forest, sweeping vegetation near river margin (4, NBM, RWC); ca. 2.5 km S of Beaver Dam, 45.7735°N, 66.6852°W, 13.VIII.2008, R. P. Webster, power-line right of way, sweeping foliage of *Alnus* sp. (10, NBM, RWC).

##### Collection and habitat data.

Adults of this species were swept from foliage along a lake margin and a river margin. Adults were defoliating *Alnus* at the site south of Beaver Dam. This species was taken during June, July, and August.

##### Distribution in Canada and Alaska.

 BC, AB, MB, ON, **NB**, NS, PE (Riley et al. 2003). This is a Palaearctic species now widely established throughout much of the northern half of the USA and Canada (Riley et al. 2003). It was introduced, together with *Neogalerucella pusilla* (Duftschmid), for the biocontrol of purple loosestrife (*Lythrum salicaria* L.) and has been successful in controlling this weed (Hight et al. 1995). Consequently, its presence on alder is incidental and the damage to the leaves may have been done before by the alder flea beetle (*Altica ambiens alni* Harris), which is closely associated with this bush (LeSage 1995).

**Map 9.**
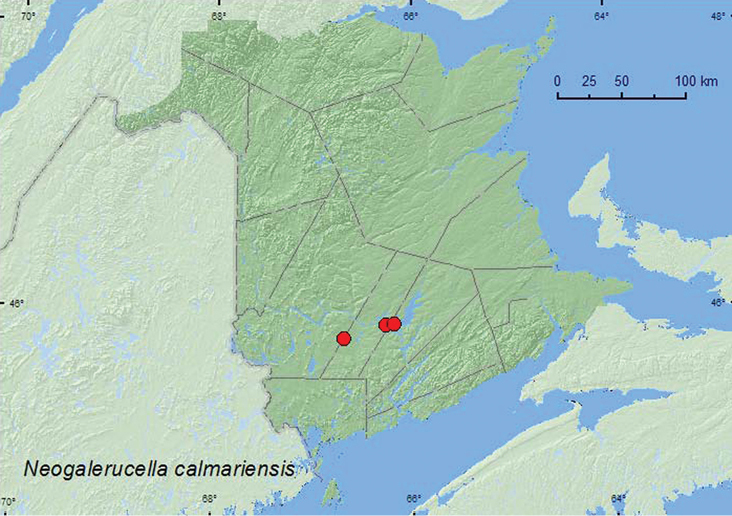
Collection localities in New Brunswick, Canada of *Neogalerucella calmariensis*.

#### Ophraella communa

LeSage, 1986**

http://species-id.net/wiki/Ophraella_communa

Map 10

##### Material examined.

**New Brunswick, Kent Co.**, Bouctouche, 20.VIII.1999, D. Audet (1, UMNB). **Sunbury Co.**, Sheffield, Portobello Creek N.W.A., 45.8950°N, 66.2728°W, 4.VIII.2004, R. P. Webster, silver maple forest, on roadside ragweed (hand picking) (9, RWC); 3.0 km SE of McGowans Corner, 45.8677°N, 66.2590°W, 6.IX.2007, R. P. Webster, silver maple forest, sweeping roadside foliage near wet meadow (ragweed present) (1, RWC).

##### Collection and habitat data.

The host plant of *Ophraella communa* is common ragweed (*Ambrosia artemisiifolia* L.), and all life stages can be found on this plant (Welch 1978). In New Brunswick, adults of *Ophraella communa* were collected from foliage of common ragweed on a roadside and swept from roadside foliage near a wet meadow in an area with ragweed. Adults were collected during August and September.

##### Distribution in Canada and Alaska.

 BC, AB, SK, ON, **NB** (LeSage 1986b).

**Map 10.**
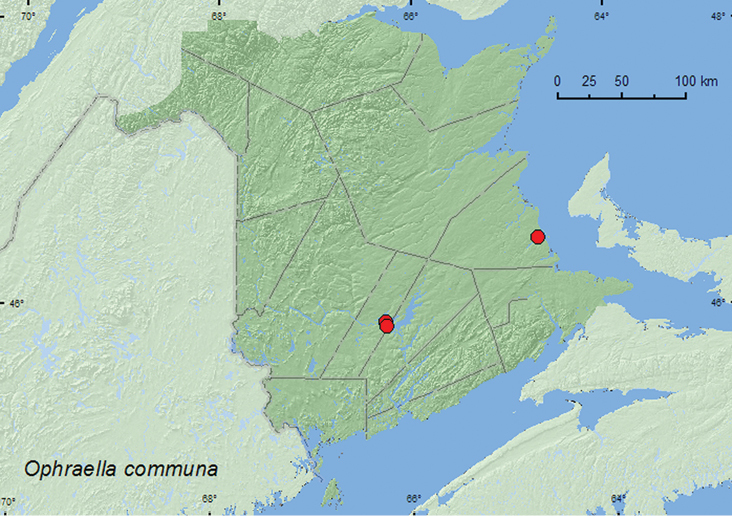
Collection localities in New Brunswick, Canada of *Ophraella communa*.

#### Ophraella cribrata (LeConte, 1865)**

http://species-id.net/wiki/Ophraella_cribrata

Map 11

##### Material examined.

**New Brunswick, Sunbury Co.**, 9.5 km NE jct Rt. 101 & 645, 45.7586°N, 66.6755°W, 22.VII.2007, 29.VII.2007, 2.VII.2008, 30.VIII.2008, R. P. Webster, old field with open sandy areas, on *Solidago* sp. (9, RWC); 7.5 km W of Tracy off Rt. 645, 45.6861°N, 66.7719°W, 26.VI.2007, R. P. Webster, old field area near roadside, on *Solidago* sp. (1, RWC).

##### Collection and habitat data.

Host plants of *Ophraella cribrata* include the goldenrods, *Solidago canadensis* L. (as *Solidago altissima* L. in LeSage 1986b), *Solidago bicolor* L., *Solidago nemoralis* Ait., *Solidago juncea* Ait., and *Solidago rugosa* P. Mill. (Fall 1924; LeSage 1986b; Clark et al. 2004), all of which occur in New Brunswick (Hinds 2000). Adults from New Brunswick were collected from *Solidago* sp. (species not determined) in an old field with open sandy areas and in an old field area near a roadside. Adults were captured during June, July, and August.

##### Distribution in Canada and Alaska.

 AB, SK, MB, ON, QC, **NB** (LeSage 1986b, 1991).

**Map 11.**
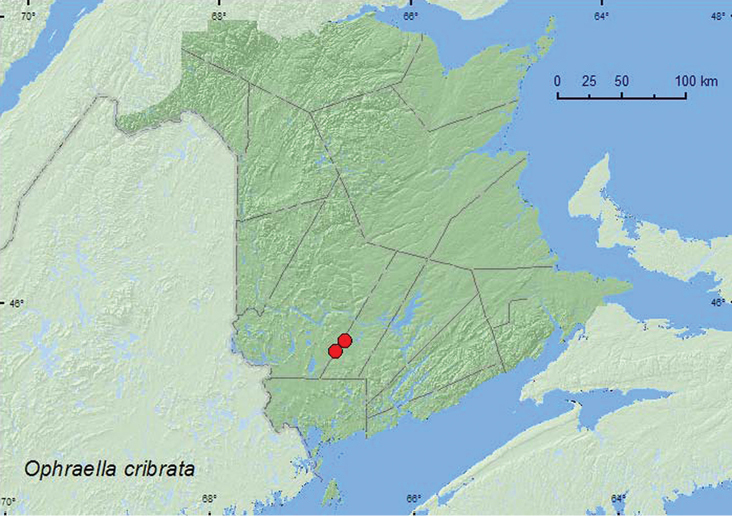
Collection localities in New Brunswick, Canada of *Ophraella cribrata*.

#### Ophraella notata

(Fabricius, 1801)**

http://species-id.net/wiki/Ophraella_notata

Map 12

##### Material examined.

**New Brunswick,** **Sunbury Co.**, 2.5 km S of Beaver Dam, 45.7735°N, 66.6852°W, 13.VIII.2008, R. P. Webster, powerline-right-of-way, sweeping (and hand picking) foliage of *Eupatorium perfoliatum* (15, NBM, RWC).

##### Collection and habitat data.

The normal host plant of *Ophraella notata* is thoroughwort or bonset (*Eupatorium perfoliatum* L.)(LeSage 1986b). Specimens from New Brunswick were abundant on this host plant in a damp meadow area along a powerline right-of-way. Adults were collected during August.

##### Distribution in Canada and Alaska.

ON, QC, **NB** (LeSage 1986b, 1991).

**Map 12.**
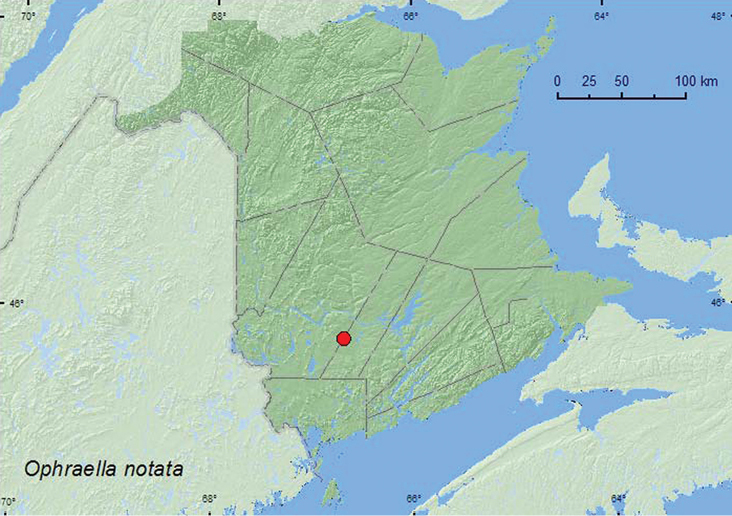
Collection localities in New Brunswick, Canada of *Ophraella notata*.

#### Tricholochmaea ribicola

(Brown, 1938)**

http://species-id.net/wiki/Tricholochmaea_ribicola

Map 13

##### Material examined.

**New Brunswick, Albert Co.**, Caledonia Gorge P.N.A., off Caledonia Mountain Rd., 45.8318°N, 64.7570°W, 1.VII.2011, R. P. Webster, small *Carex* marsh, on *Ribes* sp. (10, NBM, RWC). **Carleton Co.**, Two Mile Brook Fen, 46.3594°N, 67.6800°W, 2.VI.2005, R. P. Webster, cedar swamp, on foliage of *Ribes* sp. (10, RWC).

##### Collection and habitat data.

The New Brunswick adults were taken on wild black currant (*Ribes americanum* P. Miller) during June and July. Brown (1946) reported *Tricholochmaea ribicola* from *Ribes americanum* in other parts of its range. It has also been recorded from *Ribes vulgare* Lam. (Clark et al. 2004).

##### Distribution in Canada and Alaska.

 ON, **NB** (LeSage 1991).

**Map 13.**
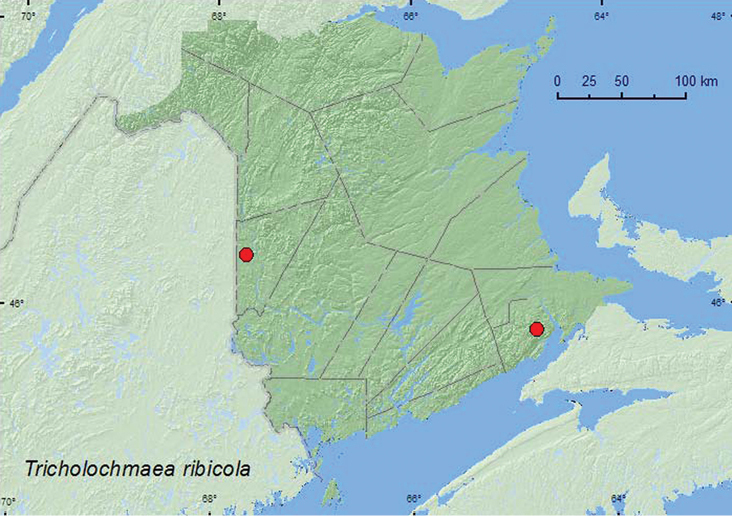
Collection localities in New Brunswick, Canada of *Tricholochmaea ribicola*.

#### Tricholochmaea rufosanguinea

(Say, 1826)**

http://species-id.net/wiki/Tricholochmaea_rufosanguinea

Map 14

##### Material examined.

**New Brunswick, York Co.**, Upper Brockway, 45.5684°N, 67.0993°W, 3.VI.2005, R. P. Webster, acid (blueberry) barrens, on foliage of *Rhododendron canadense* (10, RWC).

##### Collection and habitat data.

Adults were found on the foliage of rhodora (*Rhododendron canadense* (L.)) in a blueberry (*Vaccinium* sp.) barren during early June.

##### Distribution in Canada and Alaska.

 QC, **NB** (LeSage 1991).

**Map 14.**
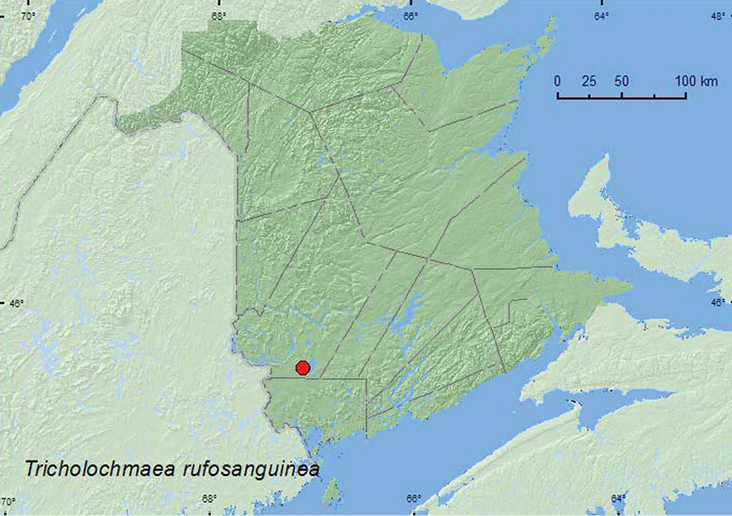
Collection localities in New Brunswick, Canada of *Tricholochmaea rufosanguinea*.

### Tribe Luperini Gistel, 1848. Subtribe Diabroticina Chapuis, 1875

#### Acalymma gouldi

Barber, 1947**

http://species-id.net/wiki/Acalymma_gouldi

Map 15

##### Material examined.

**New Brunswick, Carleton Co.**, Meduxnekeag Valley Nature Preserve, 46.1888°N, 67.6762°W, 27.VIII.2007, R. P. Webster, upper river margin, sweeping foliage of *Echinocystis lobata*, prickly cucumber (4, RWC).

##### Collection and habitat data.

Specimens of this species were swept from the foliage ofprickly cucumber(*Echinocystis lobata* (Michx.) T. & G.) along an upper river margin during August. Barber (1947) reported this species from squash (*Cucurbita*) and cucumber (*Cucumeris sativus* L.); Clark et al (2004) reported *Cucumeris melo* L. as a host.

##### Distribution in Canada and Alaska.

 ON, QC, **NB** (LeSage 1991).

**Map 16.**
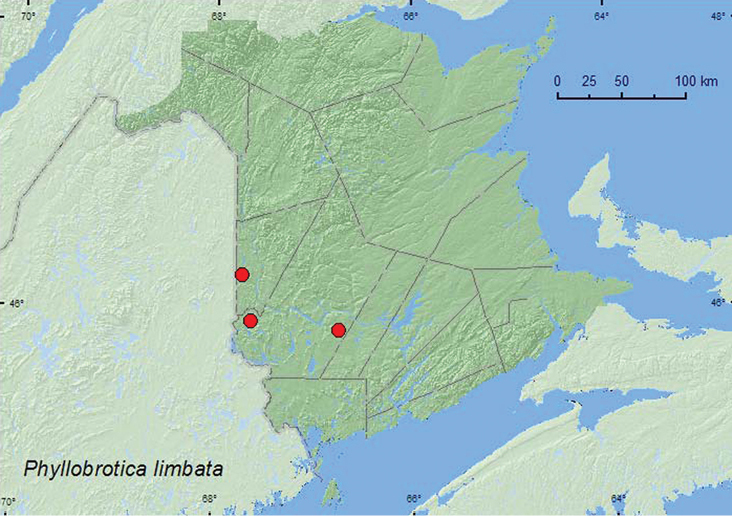
Collection localities in New Brunswick, Canada of *Phyllobrotica limbata*.

### Subtribe Luperina Chapuis, 1875

#### Phyllobrotica limbata

(Fabricius, 1801)**

http://species-id.net/wiki/Phyllobrotica_limbata

Map 16

##### Material examined.

**New Brunswick, Carleton Co.**, Jackson Falls, Bell Forest, 46.2210°N, 67.7211°W, 1.VIII.2004, 13.VIII.2007, V. Webster & R. P. Webster, mature hardwood forest, sweeping foliage (2, RWC). **Saint John Co.**, Saint John, 24.VII.1902, W. McIntosh (1, NBM). **York Co.**, Canterbury, near Browns Mountain Fen, 45.8978°N, 67.6273°W, 3.VII.2005, M.-A. Giguère & R. Webster, mixed forest, on foliage of *Corylus cornuta* (1, RWC); Charters Settlement, 45.8331°N, 66.7410°W, 11.VIII.2007, 7.VII.2008, R. P. Webster, mature red spruce and red maple forest, sweeping foliage in shaded marshy area (3, RWC).

##### Collection and habitat data.

Specimens of this species were swept from foliage in a mature hardwood forest and in a shaded marshy area in a mature red spruce (*Picea rubens* Sarg.) and red maple (*Acer rubrum* L.) forest. One individual was collected from foliage of beaked hazelnut (*Corylus cornuta* Marsh.). Hosts reported by Clark et al. (2004) occurring in New Brunswick include common skullcap (*Scutellaria galericulata* L.) and mad-dog skullcap (*Scutellaria lateriflora* L.). Adults were collected during July and August.

##### Distribution in Canada and Alaska.

 ON, QC, **NB** (LeSage 1991).

**Map 15.**
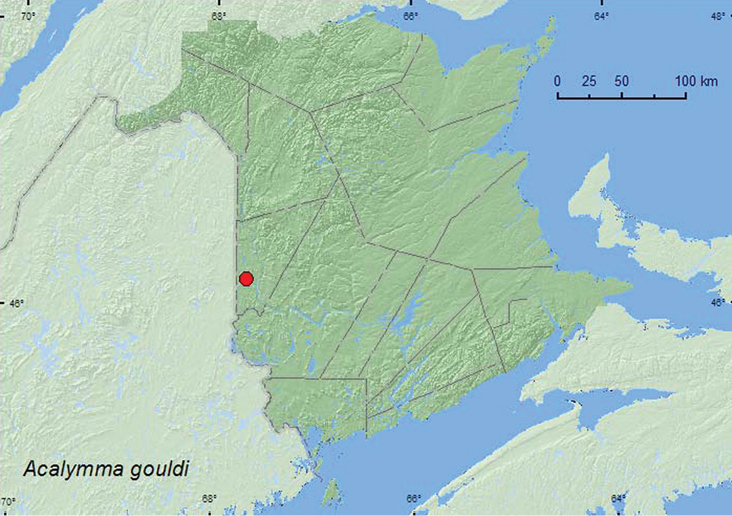
Collection localities in New Brunswick, Canada of *Acalymma gouldi*.

### Tribe Alticini Newman, 1834

#### Altica knabii

(Blatchely, 1910)**

http://species-id.net/wiki/Altica_knabii

Map 17

##### Material examined.

**New Brunswick, York Co.**, Charters Settlement, 45.8428°N, 66.7279°W, 28.IV.2004, R. P. Webster, mixed forest, in litter near small sedge marsh (1, RWC).

##### Collection and habitat data.

The only adult known from New Brunswick was sifted from leaf litter near a small *Carex* marsh during April. This was probably an overwintering site. Clark et al. (2004) reported that this species was associated with evening primrose (*Oenothera biennis* L.).

##### Distribution in Canada and Alaska.

ON, **NB** (LeSage 2008)

LeSage (2008) reported this species from Texas east to Florida and north to Minnesota and Maine in the USA.

**Map 17.**
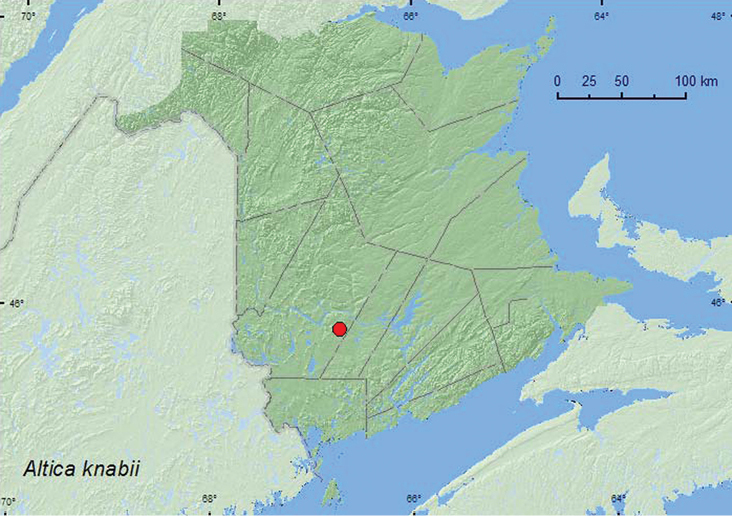
Collection localities in New Brunswick, Canada of *Altica knabii*.

#### Altica rosae

Woods, 1918**

http://species-id.net/wiki/Altica_rosae

Map 18

##### Material examined.

**New Brunswick, Carleton Co.**, Wakefield, Meduxnekeag Valley Nature Preserve, 46.1931°N, 67.6825°W, 8.VI.2005, M.-A. Giguère & R. Webster, floodplain forest, on foliage of *Rosa* sp. (1, RWC). **Queens Co.**, Grand Lake near Scotchtown, 45.8762°N, 66.1816°W, 3.VI.2007, R. P. Webster, oak / maple forest near lakeshore, sweeping foliage of *Rosa* sp. (1, RWC). **Saint John Co.**, Chance Harbour, 45.1159°N, 66.3607°W, 30.V.2006, R. P. Webster, sea beach, on foliage of *Rosa* sp. (2, RWC).

##### Collection and habitat data.

All adults from New Brunswick were collected from the foliage of *Rosa* sp., a known host for this species (Woods 1918). Adults were found during late May and early June.

##### Distribution in Canada and Alaska. 

MB, ON, QC, **NB** (Riley et al. 2003).

**Map 18.**
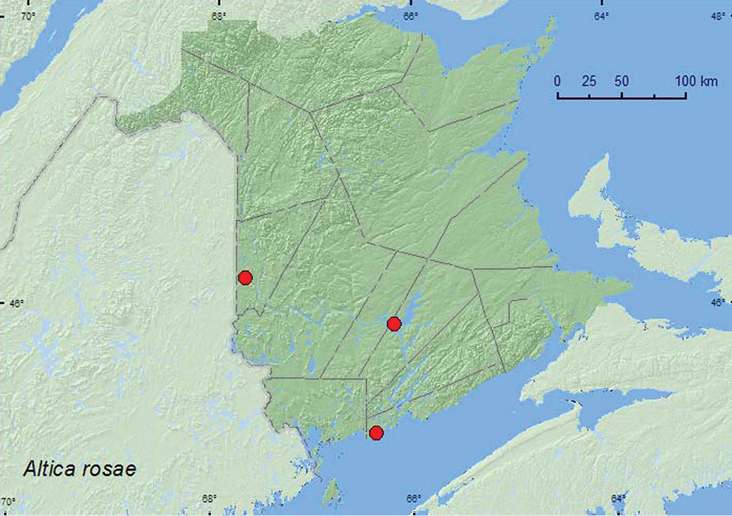
Collection localities in New Brunswick, Canada of *Altica rosae*.

#### Altica woodsi

Isely, 1920**

http://species-id.net/wiki/Altica_woodsi

Map 19

##### Material examined.

**New Brunswick, Carleton Co.**, Jackson Falls, Bell Forest, 46.2210°N, 67.7210°W, 12.VII.2004, K. Bredin, J. Edsall, & R. Webster, rich Appalachian hardwood forest, on foliage of *Vitis riparia* Michx. (4, RWC); same locality and collectors, 46.2252°N, 67.7190°W, 12.VII.2004, river margin, on foliage of *Vitis riparia* Michx. (2, NBM, RWC); same locality data, 1.VI.2005, M.-A. Giguère & R. Webster, river margin, on foliage of *Vitis riparia* Michx. (3, RWC); Meduxnekeag Valley Nature Preserve, 46.1925°N, 67.6725°W, 13.VII.2005, R. P. Webster, mixed forest, on foliage of *Vitis riparia* Michx. (1, RWC).

##### Collection and habitat data.

*Altica woodsi* was collected from the foliage of river bank or frost grape (*Vitis riparia* Michx.) in a rich Appalachian hardwood forest, a mixed forest, and along river margins in New Brunswick. Adults were collected during June and July. The Virginia creeper (*Parthenocissus quinquefolia* (L.) Planch.) is an alternate host used by both the larvae and adults (LeSage and Zmudzinska 2004).

##### Distribution in Canada and Alaska. 

ON, QC, **NB** (LeSage 2002; Riley et al. 2003).

**Map 19.**
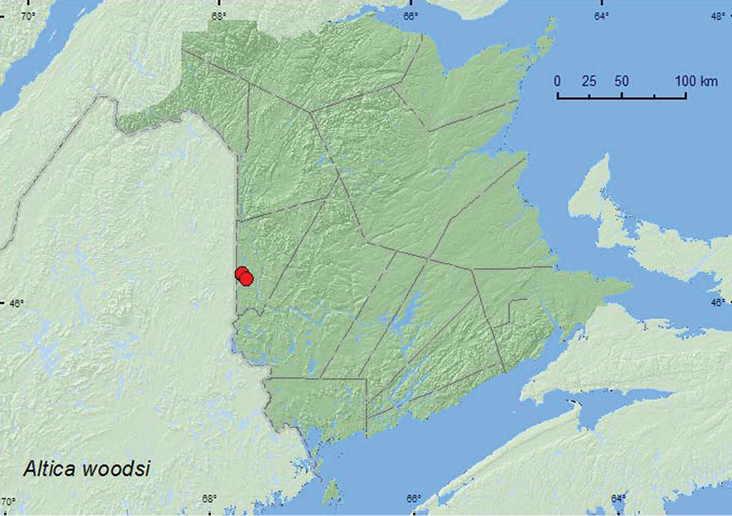
Collection localities in New Brunswick, Canada of *Altica woodsi*.

#### Crepidodera violacea

Melsheimer, 1847**

http://species-id.net/wiki/Crepidodera_violacea

Map 20

##### Material examined.

**New Brunswick, Carleton Co.**, Meduxnekeag Valley Nature Preserve, 46.1890°N, 67.6766°W, 8.VI.2005, M.-A. Giguère & R. Webster, flood plain forest, beating foliage of *Prunus virginiana* (10, RWC).

##### Collection and habitat data.

Parry (1986) reported *Crepidodera violacea*from *Crataegu*s and *Prunus*, including choke cherry (*Prunus virginiana* L.). Other host plants reported by Clark et al. (2004) known to occur in New Brunswick are *Amelanchier*, pin cherry (*Prunus pensylvanica* L.), and black cherry (*Prunus serotina* Ehrh.). Adults from New Brunswick were collected by beating foliage of choke cherry during June.

##### Distribution in Canada and Alaska.

 ON, QC, **NB** (LeSage 1991).

**Map 20.**
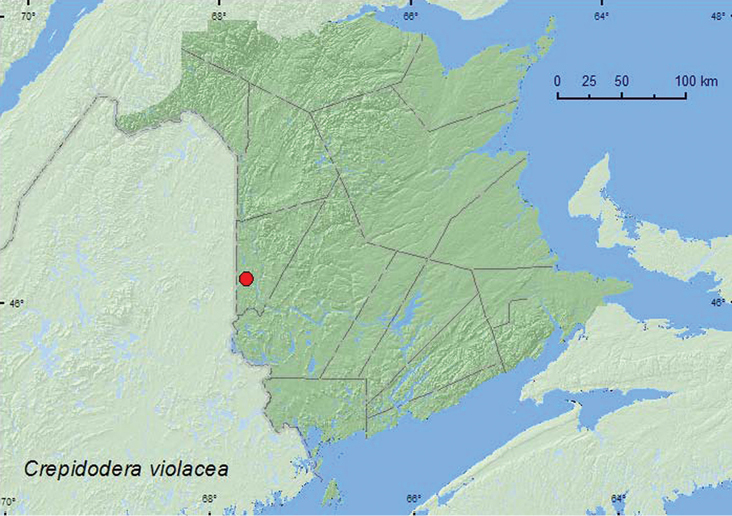
Collection localities in New Brunswick, Canada of *Crepidodera violacea*.

#### Longitarsus erro

Horn, 1889

http://species-id.net/wiki/Longitarsus_erro

Map 21

##### Material examined.

**New Brunswick, Saint John Co.**, Dipper Harbour, 45.1169°N, 66.3771°W, 12. IX.2006, R. P. Webster, sea beach, sweeping vegetation (1, RWC).

##### Collection and habitat data. 

One individual of this species was swept from foliage along a sea beach during September.

##### Distribution in Canada and Alaska.

 NT, BC, AB, MB, ON, QC, **NB**, NS (LeSage 1991).

**Map 21.**
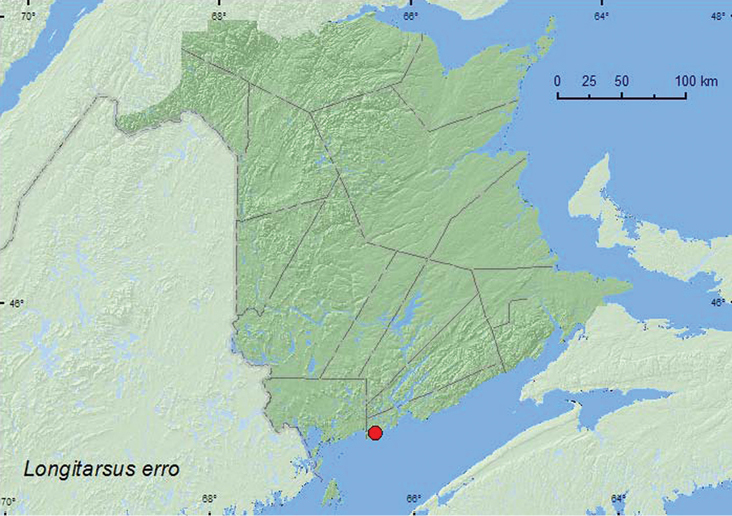
Collection localities in New Brunswick, Canada of *Longitarsus erro*.

#### Mantura chrysanthami

(Koch, 1803)

http://species-id.net/wiki/Mantura_chrysanthami

Map 22

##### Material examined.

**New Brunswick, Charlotte Co.**, near Maces Bay, 45.12447°N, 66.47346°W, 12.VIII.2007, R. P. Webster, barrier beach, sweeping vegetation (1, RWC). **Northumberland Co.**, Blueberry Rd. off Hwy 8, 47.3211°N, 65.4229°W, 29.VI.2007, R. P. Webster, jack pine forest with black spruce, sweeping foliage of *Rumex acetosella* L. (4, RWC). **Queens Co.**, Canning, Grand Lake near Scotchtown, 45.8762°N, 66.1816°W, 1.VII.2004, D. Sabine & R. Webster, lake shore, old dune with oaks, sweeping foliage (3, RWC). **Sunbury Co.**, ca. 2.5 km S of Beaver Dam, 45.7703°N, 66.6867°W, 26.VI.2007, mixed forest with red pine, along power-line cut, sweeping foliage (1, RWC). **York Co.**, Canterbury, near “Browns Mtn. Fen”, 45.8978°N, 67.6273°W, 3.VII.2005, M.-A. Giguère & R. Webster, mixed forest, beating foliage (1, RWC).

##### Collection and habitat data.

*Mantura chrysanthami* was swept or beaten from foliage from a variety of habitats in New Brunswick. These included a barrier beach, a jack pine forest, an old sand dune with red oaks (*Quercus rubra* L.), a power-line right-of-way, and a mixed forest. A small series was swept from the foliage of sheep sorrel, *Rumex acetosella* L. Adults were captured during June, July, and August. Based on personal observations and collecting by the second author in the Ottawa, ON area, *Mantura chrysanthemi* is monophagous on *Rumex acetosella* both in the larval and adult stages.

##### Distribution in Canada and Alaska.

 NF, QC, **NB** (LeSage 1991; Riley et al. 2003). This is an adventive Palaearctic species now established in most of the northeastern United States (Riley et al. 2003). Although *Mantura floridana* Crotch was cited by LeSage (1991) and Riley et al. (2003) from the Maritime provinces, the specimens determined as this species may be *Mantura chrysanthemi*, and thus the status of the former needs to be clarified.

**Map 22.**
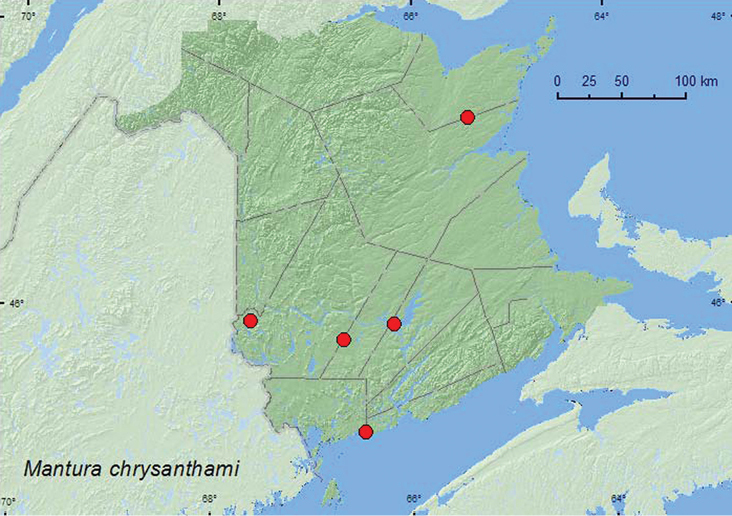
Collection localities in New Brunswick, Canada of *Mantura chrysanthami*.

#### Psylliodes affinis

(Paykull, 1799)**

http://species-id.net/wiki/Psylliodes_affinis

Map 23

##### Material examined.

**New Brunswick, Charlotte Co.**, near Maces Bay, 45.12447°N, 66.47346°W, 12.VIII.2007, R. P. Webster, barrier beach, sweeping *Solanum* sp. (10, RWC).

##### Collection and habitat data.

A series of *Psylliodes affinis* from New Brunswick was swept from the foliage of a *Solanum* sp. on a barrier beach during August. The second author observed leaves of the climbing nightshade (*Solanum dulcamara* L.) in Aylmer (QC), north of Ottawa (ON), punctured with many small holes by adults of *Psylliodes affinis*.

##### Distribution in Canada and Alaska.

 ON, QC, **NB** (LeSage 1991; Riley et al. 2003). This is an adventive Palaearctic species now established in most of the northeastern United States (Riley et al. 2003).

**Map 23.**
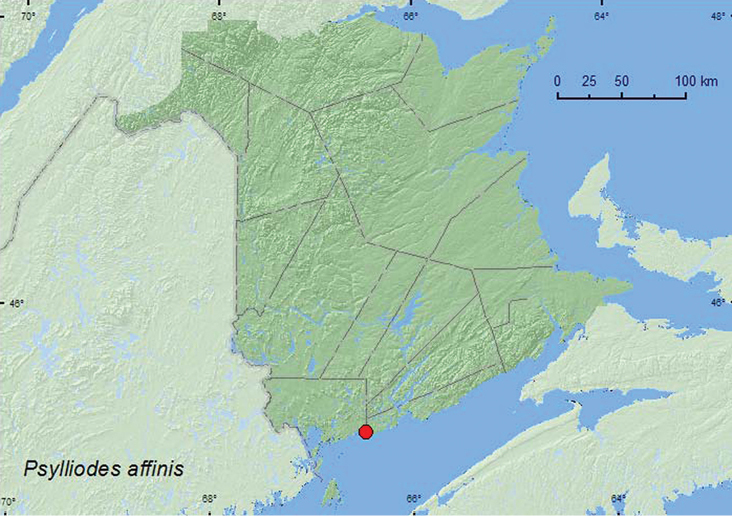
Collection localities in New Brunswick, Canada of *Psylliodes affinis*.

#### Systena hudsonias

(Forster, 1771)**

http://species-id.net/wiki/Systena_hudsonias

Map 24

##### Material examined.

**New Brunswick, Northumberland Co.**, Blueberry Rd. off Hwy 8, 47.3210°N, 65.4229°W, 24.VII.2005, R. P. Webster, jack pine forest, sweeping(1, RWC). **York Co.**, Charters Settlement, 45.8430°N, 66.7275°W, 27.VI.2004, 17.VII.2007, 30.VI.2008, R. P. Webster, regenerating mixed forest in brushy opening, sweeping foliage (4, RWC); Canterbury, near “Browns Mtn. Fen”, 45.8978°N, 67.6273°W, 3.VII.2005, M.-A. Giguère & R. Webster, mixed forest, beating foliage (on roadside) (1, RWC).

##### Collection and habitat data.

This is a polyphagous species reported from hosts in 19 families (Clark et al. 2004). Most adults of *Systena hudsonias* from New Brunswick were swept from foliage in old field habitats. Adults were captured during July.

##### Distribution in Canada and Alaska.

 MB, ON, QC, **NB** (LeSage 1991).

**Map 24.**
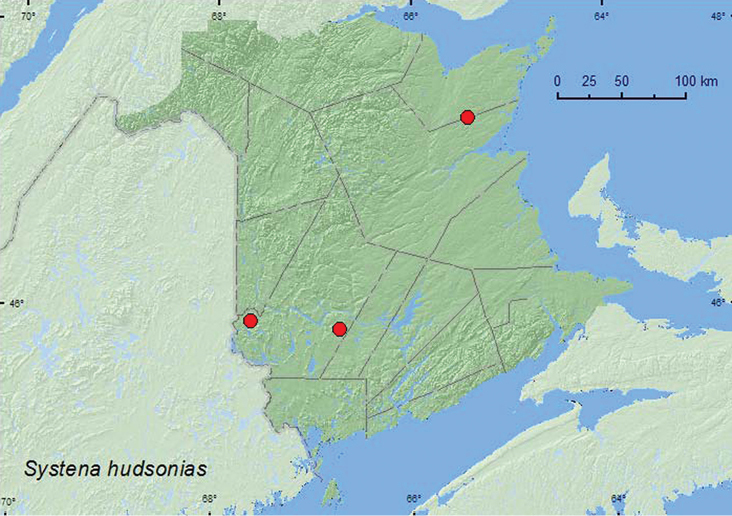
Collection localities in New Brunswick, Canada of *Systena hudsonias*.

### Subfamily Cryptocephalinae Gyllenhal, 1813. Tribe Cryptocephalini Gyllenhal, 1813. Subtribe Pachybrachina Chapius, 1874

#### Pachybrachis bivittatus

(Say, 1824)**

http://species-id.net/wiki/Pachybrachis_bivittatus

Map 25

##### Material examined.

**New Brunswick, Restigouche Co.**, Jacquet River Gorge P.N.A., (at the Jacquet River) 47.8197°N, 66.0835°W, 23.VI.2008, D. McAlpine & R. Webster, river margin, on *Salix* foliage (20, CNC, NBM, RWC).

##### Collection and habitat data.

Adults of this species were abundant on *Salix* foliage along a river margin during June. LeSage (1985) reared the larvae on decaying leaves of willow.

##### Distribution in Canada and Alaska.

 BC, AB, SK, ON, QC, **NB** (LeSage 1991).

**Map 25.**
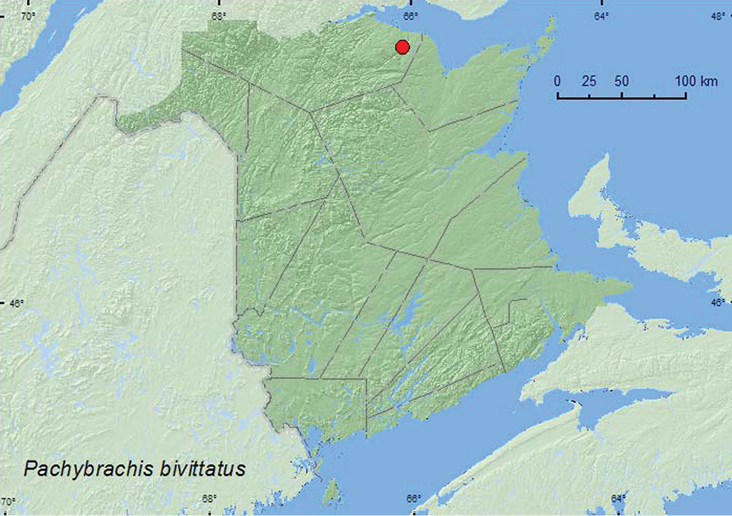
Collection localities in New Brunswick, Canada of *Pachybrachis bivittatus*.

#### Pachybrachis m-nigrum

(Melsheimer, 1847)**

http://species-id.net/wiki/Pachybrachis_m-nigrum

Map 26

##### Material examined.

**New Brunswick,** **York Co.**, 15.0 km W of Tracy off Rt. 645, 45.6837°N, 66.8809°W, 22.VII.2007, R. P. Webster, old red pine forest, sweeping foliage of *Comptonia peregrina* (2, CNC, RWC).

##### Collection and habitat data.

Two individuals were swept from foliage of sweet-fern (*Comptonia peregrina* (L.)) near an old red pine forest during July.

##### Distribution in Canada and Alaska. 

QC, **NB** (LeSage 1991).

**Map 26.**
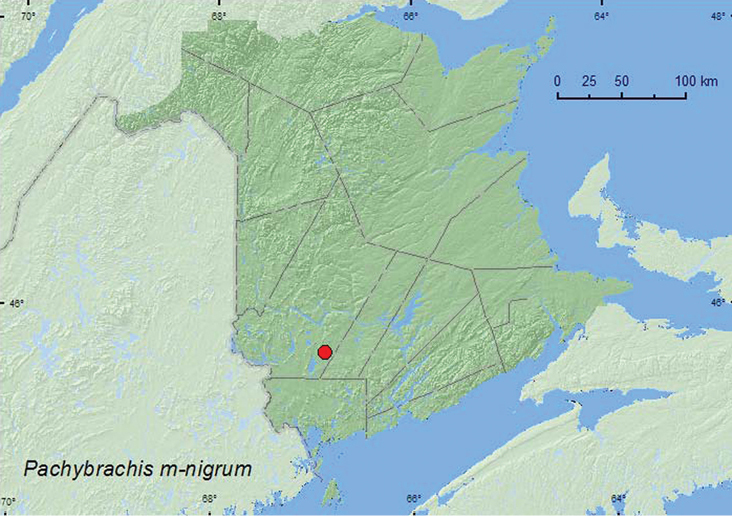
Collection localities in New Brunswick, Canada of *Pachybrachis m-nigrum*.

### Subtribe Cryptocephalina Gyllenhal, 1813

#### Bassareus formosus

(Melsheimer, 1847)

http://species-id.net/wiki/Bassareus_formosus

Map 27

##### Material examined.

**New Brunswick, Gloucester Co.**, Airstrip off Hwy 8, 47.3330°N, 65.4282°W, 24.VII.2005, R. P. Webster, jack pine/spruce forest, on foliage of *Comptonia peregrina* (4, RWC). **Northumberland Co.**, Blueberry Rd. off Hwy 8, 47.3210°N, 65.4229°W, 24.VII.2005, R. P. Webster, jack pine forest, on foliage of *Comptonia peregrina* (7, RWC). **Sunbury Co.**, 9.5 km NE jct Rt. 101 & 645, 45.7586°N, 66.6755°W, 17.VII.2008, R. P. Webster, old field with open sandy areas, sweeping foliage (1, RWC); 2.5 km S of Beaver Dam, 45.7735°N, 66.6852°W, 13.VIII.2008, R. P. Webster, powerline-right-of-way, sweeping foliage of *Comptonia peregrina* (1, RWC). **York Co.**, Charters Settlement, 45.8430°N, 66.7275°W, 20.VII.2008, R. P. Webster, old field area in regenerating mixed forest, sweeping foliage (1, RWC).

##### Collection and habitat data. 

Most adults of *Bassareus formosus* in New Brunswick were swept from foliage of *Comptonia peregrina* in old fields and other forest openings during July and August. The repeated collection of *Bassareus formosus* fromthis plant suggests a close association with it that was not reported by Clark et al. (2004).

##### Distribution in Canada and Alaska.

ON, QC, **NB**, NS (LeSage 1991).

**Map 27.**
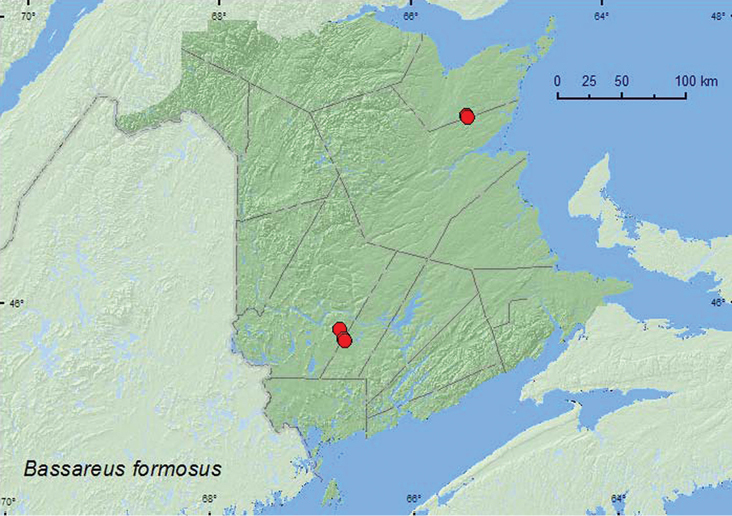
Collection localities in New Brunswick, Canada of *Bassareus formosus*.

#### Bassareus mammifer

(Newman, 1840)**

http://species-id.net/wiki/Bassareus_mammifer

Map 28

##### Material examined.

**New Brunswick, Kent Co.**, Kouchibouguac National Park, 7.VII.1970, H. Goulet, 7785K (1, CNC); same locality, 1.VIII.1978, D. B. Lyons, 7400P (1, CNC). **Madawaska Co.**, Edmundston, 19.VII.1970, C. M. Yoshimoto (2, CNC). **Northumberland Co.**, Boisetown, 10.VII.1928, W. J. Brown (1, CNC); 2 mi Bradlebane (sic) (Breadalbane) Rd., 11.VII.1966 (R. M. Smith), on white birch, 66–1907–02 (1, AFC). **Queens Co.**, Chipman, Harley Rd., 22.VI.1987 (D. H. Clark), on *Acer rubrum*, 87–2284–03 (1, RFC). **Restigouche Co.,** Indian Brook, (on NW Upsalquitch) 5.VII.1976 (Edward Belliveau), on trembling aspen, 76–2-3358–05 (2, CNC, AFC). **York Co.**, Durham, 8.VII.1956, G. W. Barter, on *Populus tremuloides* (1, AFC).

##### Collection and habitat data.

Adults of *Bassareus mammifer* from New Brunswick were collected from foliage of trembling aspen (*Populus tremuloides* Michx.), white birch (*Betula papyrifera* Marsh.), and red maple during June, July, and August.

##### Distribution in Canada and Alaska.

AB, MB**,** ON, QC, **NB** (LeSage 1991; Riley et al. 2003).

**Map 28.**
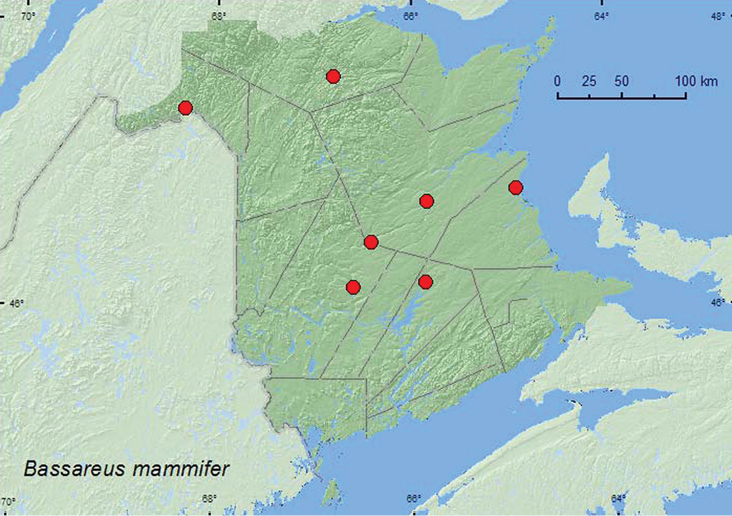
Collection localities in New Brunswick, Canada of *Bassareus mammifer*.

#### Cryptocephalus venustus

Fabricius, 1787**

http://species-id.net/wiki/Cryptocephalus_venustus

Map 29

##### Material examined.

**New Brunswick, Sunbury Co.**, 9.5 km NE jct. Rt. 101 & 645, 45.7586°N, 66.6755°W, 17.VII.2008, R. P. Webster, old field with open sandy areas, sweeping foliage (3, RWC). **York Co.**, Charters Settlement, 45.8340°N, 66.7450°W, 10.VII.2005, R. P. Webster, old field, sweeping (3, RWC); same locality but 45.8430°N, 66.7275°W, 17.VIII.2007, R. P. Webster, regenerating mixed forest, sweeping foliage in brushy opening (1, RWC).

##### Collection and habitat data.

This is a polyphagous species reported from hosts in 13 families (Clark et al. 2004). *Cryptocephalus venustus* was collected by sweeping foliage in an old field with sandy areas, a small old-field opening in a mixed forest, and in a brushy opening within a 20-year-old regenerating mixed forest. Adults were captured during July and August. LeSage (1986) successfully reared the larvae of this species on a mixture of dead leaves of *Alnus*, *Rubus*, *Salix*, and *Vaccinium* spp.

##### Distribution in Canada and Alaska.

 AB, SK, MB, ON, QC, **NB** (LeSage 1991; Riley et al. 2003).

**Map 29.**
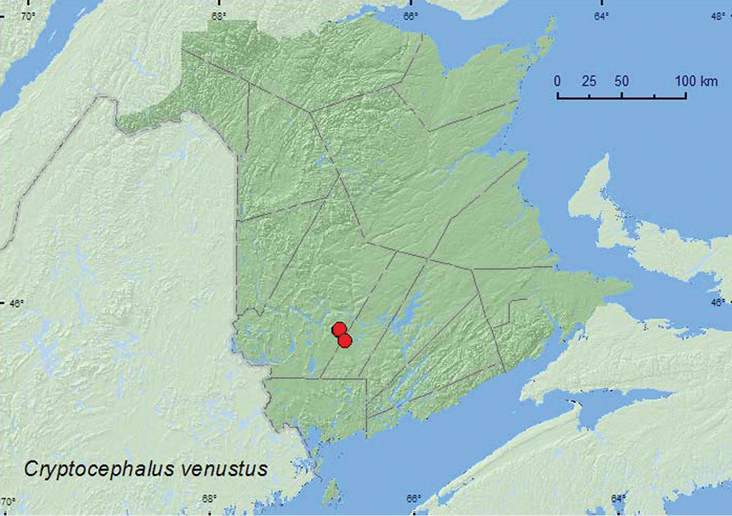
Collection localities in New Brunswick, Canada of *Cryptocephalus venustus*.

## Supplementary Material

XML Treatment for Zeugophora varians

XML Treatment for Neohaemonia melsheimeri

XML Treatment for Neohaemonia nigricornis

XML Treatment for Odontota dorsalis

XML Treatment for Chrysolina marginata

XML Treatment for Chrysomela laurentia

XML Treatment for Prasocuris vittatus

XML Treatment for Erynephala maritima

XML Treatment for Neogalerucella calmariensis

XML Treatment for Ophraella communa

XML Treatment for Ophraella cribrata

XML Treatment for Ophraella notata

XML Treatment for Tricholochmaea ribicola

XML Treatment for Tricholochmaea rufosanguinea

XML Treatment for Acalymma gouldi

XML Treatment for Phyllobrotica limbata

XML Treatment for Altica knabii

XML Treatment for Altica rosae

XML Treatment for Altica woodsi

XML Treatment for Crepidodera violacea

XML Treatment for Longitarsus erro

XML Treatment for Mantura chrysanthami

XML Treatment for Psylliodes affinis

XML Treatment for Systena hudsonias

XML Treatment for Pachybrachis bivittatus

XML Treatment for Pachybrachis m-nigrum

XML Treatment for Bassareus formosus

XML Treatment for Bassareus mammifer

XML Treatment for Cryptocephalus venustus
